# Pupil light reflex in young elite athletes: autonomic nervous system activity and viscoelastic properties

**DOI:** 10.3389/fphys.2024.1421676

**Published:** 2024-07-30

**Authors:** Cyril J. P. Giovannangeli, Fabio Borrani, Olivier Broussouloux, Olivier Maurelli, Laurent Schmitt, Robin B. Candau

**Affiliations:** ^1^ DMeM, INRAE, University of Montpellier, Montpellier, France; ^2^ Corsican Center for Sport and Youth, Ajaccio, France; ^3^ Department of Biomedical Sciences, University of Lausanne, Lausanne, Switzerland; ^4^ P3S Unit, University of Corsica, Corte, France; ^5^ Professor Emeritus of National School of Mountain Sports/National Ski-Nordic Centre, Premanon, France

**Keywords:** photomotor reflex, sympathetic nervous system, parasympathetic nervous system, smooth muscle stiffness, monitoring

## Abstract

**Introduction:** The pupil light reflex (photomotor reflex) has a duration of 3.5 s and is a highly reproducible measurement. Conventionally, the autonomic nervous system (ANS) activity evaluated by this reflex does not consider the viscoelasticity of the iris muscles. This study aims to detect differences in reflex autonomic activity in a supine position with parameters derived from the Kelvin-Voigt viscoelastic model in two distinct groups of elite athletes.

**Method:** Groups formed using a dendrogram analysis based on basal autonomic activity assessed with heart rate variability. Heart rate variability was measured, and the photomotor reflex was modeled.

**Results:** The model showed a high degree of adjustment to the photomotor reflex (r^2^ = 0.99 ± 0.01). The impulse 3, an indicator of reflex sympathetic activity, revealed a significantly higher activity (ρ ≤ 0.05) in the [sympa/para]^+^ group compared to the [sympa/para]^⁻^ group. This result was further supported by a greater relative total redilation amplitude (ρ ≤ 0.05) and a shorter duration of 75% redilation (ρ ≤ 0.01). Finally, the relative total redilation amplitude exhibited a significant correlation with the linear stiffness constant (ρ ≤ 0.001) and the maximum redilation speed with restoring force (ρ ≤ 0.001).

**Discussion:** These results indicate that (i) the photomotor reflex can detect an alteration of the reflex autonomic activity specific to each of the two branches of the ANS (ii) the viscoelastic properties of the iris muscles play a significant role in the energy storage-restitution mechanisms during the photomotor reflex. This approach could allow athletes to benefit from reduced time spent in the analysis of ANS activity, potentially making it an almost daily and automated process.

## Introduction

The state of the autonomic nervous system (ANS) activity, analyzed in a supine position, including parameters such as root mean square of successive differences (RMSSD) and mean heart rate, has exhibited a close correlation with performance. This correlation was observed in the context of the championship podium appearances achieved per year in the World Cup by the most decorated male biathlete from 2009 to 2019 (Schmitt et al., 2021). The study by Pichot (Pichot et al., 2000) on middle-distance runners ranked at the national level in France shows that 3 weeks of heavy training period increases nocturnal mean heart rate parameters and decreases high frequencies (HF) compared to the following 4th week of relative resting period, suggesting the existence of close relationships between the state of the ANS, performance, and the athlete’s fatigue level.

Research has demonstrated a correlation between parameters related to heart rate variability (HRV) in healthy individuals and parameters associated with the pupil light reflex, also known as the photomotor reflex; both allow the assessment of autonomic activity (Kaltsatou et al., 2011; Okutucu et al., 2016). The photomotor reflex, collected for a duration of 3.5 s, represents a highly reproducible measure, as a series of 12 reflexes in 13 young adults almost completely overlap with each other (Cuve et al., 2022). Lowenstein and Loewenfeld’s 1950 studies (Lowenstein and Loewenfeld, 1950a; Lowenstein and Loewenfeld, 1950b) on sympathectomized and parasympathectomized animals serve as a reference for understanding the biological significance of each phase of the photomotor reflex and shed light on their individual significance.

Endurance athletes, known to have a higher basal parasympathetic activity than sedentary subjects or athletes in sports requiring strength and explosiveness like gymnastics, show a longer duration of constriction in the first phase of the reflex (Capão Filipe et al., 2003). A period of combined stress from physical exercises equivalent to 8–10,000 kcal/day, sleep deprivation and caloric deficit led to a decrease constriction amplitude compared to a resting state in military personnel (Karlsen and Søli, 1979); whereas the significant basal parasympathetic activity in endurance athletes leads to a higher constriction amplitude compared to control subjects (Capão Filipe et al., 2003). This basal parasympathetic activity in endurance athletes increases the duration of 75% redilation (Capão Filipe et al., 2003).

However, studies do not distinguish the viscoelastic forces, inherent in iris muscles activity (Yamaji et al., 2003), from those specifically attributed to the parasympathetic and sympathetic nervous systems (Lowenstein and Loewenfeld, 1950a; Lowenstein and Loewenfeld, 1950b). The photomotor reflex is not only the result of a sequence of successive reflex activations of forces from the parasympathetic and sympathetic branches of the ANS but the result of a combination of these with viscoelastic forces. In 1995, Usui and Hirata (Usui and Hirata, 1995) first integrated these viscoelastic properties into a model of the photomotor reflex. However, this model was very complex, with a total of 19 equations, making its application difficult. More recently, two studies conducted by engineers (Fan and Yao, 2011; Yan et al., 2021) integrated viscous forces (Yamaji et al., 2003), elastic forces (or restoring forces) (Heys and Barocas, 1999; Yamaji et al., 2003), and forces from the two branches of the ANS (Lowenstein and Loewenfeld, 1950a; Lowenstein and Loewenfeld, 1950b) underlying the photomotor reflex thanks to a much more applicable validated mathematical modeling with the Kelvin-Voigt model. These latest advances have integrated fundamental physical components of the photomotor reflex and are distinct from the first servo-analytic models (Stark and Sherman, 1957) or primarily descriptive models (Longtin and Milton, 1989).

In this context, we formulate three hypotheses. First, we hypothesize that the model developed by Yan et al. (2021) applied to a population of sedentary individuals and subjects with diabetes mellitus, can be utilized to characterize the state of the autonomic nervous system in young elite athletes by taking into consideration the viscoelastic properties of the iris muscles, i.e., the dilator and constrictor muscles. Our second hypothesis is that the Kelvin-Voigt model is capable of detecting subtle differences in the respective activities of the two branches of the ANS between two groups belonging to a population of young elite athletes. Our third hypothesis suggests that the reflex autonomic activity evaluated with the Kelvin-Voigt model applied to the photomotor reflex not only yields analogous insights to those derived from basal autonomic activity assessed through HRV but also offers novel complementary information.

## Materials and methods

### Experimental approach to the problem

**FIGURE 1:**
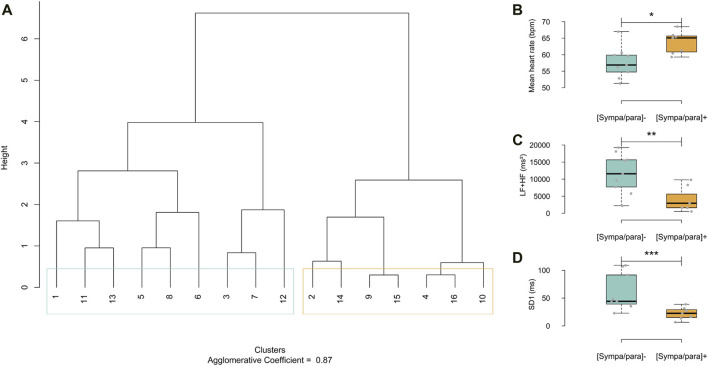
**(A)** Dendrogram displaying HRV parameters, mean heart rate in beats per minute (bpm), low frequencies + high frequencies (LF + HF) and standard deviation 1 (SD1). In light blue, group with low sympathetic/parasympathetic ratio [(sympa/para)^⁻^] and in orange, group with high sympathetic/parasympathetic ratio [(sympa/para)^+^]. **(B–D)** Graphical representation of HRV variables used in the dendrogram analysis. [sympa/para]- group (HRV = 9) and [sympa/para]+ group (n HRV = 7) *ρ ≤ 0.05, **ρ ≤ 0.01, *** ≤0.001.

As proposed by Bourdillon (Bourdillon et al., 2022), this study employed analyses within the temporal and frequency domains, augmented by a non-linear domain analysis (Guzik et al., 2007). Two groups of athletes with distinct basal sympathetic/parasympathetic activities were formed based on a dendrogram analysis (Figure 1A); the agglomerative coefficient was 0.87 (range from 0 to 1). The agglomerative coefficient measures how well subjects can be grouped based on their similar properties regarding the analyzed constants. This coefficient reflects the cohesion within the formed groups: a value close to one indicates a high similarity within groups, while a value closer to 0 suggests more marked heterogeneity. This method allows for the grouping of subjects with similar properties, leading to the formation of groups that are not necessarily of equal size. The distance between the branches of the dendrogram was calculated using Ward’s aggregation method, enabling a hierarchical classification based on the minimization of within-group variance and maximizing the variance between groups. The panels in Figures 1B–D illustrate the disparities among the parameters used in the dendrogram analysis to categorize the athletes into two groups: mean heart rate in beats per minute (bpm), the total spectral power of the frequency bands Low Frequencies + High Frequencies (LF + HF) in milliseconds squared (ms^2^) (Schmitt et al., 2015), which is the integrated measure of power spectral density in ms^2^/Hz, and standard deviation 1 (SD1) in milliseconds (ms) (Guzik et al., 2007), which is the measure of the standard deviation of the distances of the points from a line perpendicular to the line of identity in the Poincaré analysis. A group of nine athletes with a relatively low basal sympathetic/parasympathetic ratio [(sympa/para)^⁻^: blue group] and a group of seven athletes with a relatively high basal sympathetic/parasympathetic ratio [(sympa/para)^+^: orange group] were identified.

### Subjects

The participants consisted of sixteen male handball and basketball players (n = 9, n = 7) with the following characteristics (mean ± SD): age = 15.9 ± 0.9 years; weight = 72.8 ± 9.1 kg; height = 1.82 ± 0.07 m). These athletes were in their postseason recovery phase and had maintained a high-level sports regimen for at least 1 year. All participants had either normal vision or vision corrected to normalcy. The tests were conducted between 08:00 and 13:00 at sea level. The recording of photomotor reflex and HRV was done during the same period. Athletes were instructed to avoid spending any time at high altitudes in the 7 days preceding the tests (Wilson et al., 2008). They were instructed not to consume caffeine-based substances (Abokyi et al., 2017) and to refrain from intense physical and mental exercises (Karlsen and Søli, 1979) 24 h before the start of the protocol. Athletes and their parents received comprehensive information regarding the study’s objectives, potential benefits, associated risks, and procedures. They read and provided their signed informed consent to participate in the study. All procedures were conducted in accordance with the Helsinki Declaration, and ethical approval for the study was granted by the University of Montpellier Research Ethics Committee (UM 2024-009bis).

### Procedures

#### Heart rate variability

The analysis procedures and data processing of HRV were carried out following the recommendations (Heart rate Variability, 1996; Bourdillon et al., 2017). Throughout the protocol, athletes wore a chest heart rate monitor (Polar^®^ H9 by Polar—Finland) to measure the durations of intervals between each R wave of cardiac electrical activity. Signals were recorded using the EliteHRV 5.5.6 application on iPhone 11 and exported to Kubios^®^ HRV standard 3.5.0 for analysis, indicated as a valid method of HRV analysis in athletes while in a supine position (Perrotta et al., 2017). After spending 5 min in the room for equipment setup, the athletes lay down, and the 5 min HRV recording began after a 1-min period in a supine position on their backs. Within this 5-min analysis window, data analyses are based on 4 min of RR intervals, from the first to the fifth minute; the first minute allowing for a regular respiratory rhythm so that respiratory sinus arrhythmia does not drift during the HRV test and does not lead to a misinterpretation of the frequency domain parameters (Saboul et al., 2012). Ectopic beats in the 4-min RR interval recordings were corrected using threshold very low automatic inspections proposed by Kubios. From the RR intervals, temporal, frequency, and non-linear HRV parameters were extracted and included mean heart rate in bpm, RMSSD in ms, standard deviation of normal-to-normal intervals (SDNN) in ms, HF in ms^2^ (0.15–0.40 Hz), LF + HF in ms^2^, the LF/HF ratio (unitless) (Heart rate Variability, 1996), standard deviation 1 (SD1), the ratio of standard deviation 2 over standard deviation 1 (SD2/SD1) (Guzik et al., 2007), and the detrended fluctuation analysis alpha 1 (DFAα1) (Beckers et al., 2006).

#### Photomotor reflex

All athletes individually entered the specific experimental room to equip themselves and adapt to the ambient light for 5 min before lying down. They were placed in a supine position on their backs, with their heads turned towards the television screen. The room was soundproof, windowless, and maintained at a constant temperature of approximately 24°C. The walls and floor were covered in black. The basal illumination perpendicular to the center of the turned-on black screen, whose brightness was set to 0 at a distance of 55 cm from the athlete’s forehead (Wang et al., 2018), was adjusted to a value of 10 lux (Bhowmik et al., 2021). This measurement was made using a luxmeter (Lightmeter turbotech TT1308–China). The lighting was provided by three halogen lamps equipped with a dimmer with a color temperature of about 3,000 K. The athletes’ gaze was fixed on the central area of the television screen (Samsung QB50R LED 50 inches—Vietnam) which generated light flashes. After a 1-min adaptation phase in the supine position, the start analysis began with ten 200 ms flashes, each spaced 30 s apart, for a total test duration of 5 min. The screen was continuously active during the adaptation phase and between flashes. Participants were required to minimize eye movements to avoid variations in accommodation and angle of vergence and to refrain from blinking during and a few seconds after each light flash. These flashes, with an intensity of 30 lux (Bhowmik et al., 2021), were red. The color temperature and gamma number BT 1886 were set to 7,000 K and −5, respectively. The flash conditions and their timing were regulated by a Python-written program. Athletes wore glasses equipped with two cameras (Pupil core by Pupil Labs–Berlin, Germany) to record the photomotor reflex at a sampling frequency of 120 Hz. This device was validated against a reference system (Ehinger et al., 2019). The classical descriptive analysis of the photomotor reflex was performed on Rstudio 2022.07.1 to extract the different parameters. The duration of latency is defined as the time interval between the start of the light stimulus and the beginning of the iris constrictor contraction (Kaltsatou et al., 2011). The onset of iris constrictor contraction was defined at the moment when the constriction speed exceeded 0.40 mm/ms. Constriction amplitude is defined as the difference between the basal diameter and the minimum diameter, diameter at 0 s−minimum diameter in mm (Kaltsatou et al., 2011). This parameter allowed for the calculation of the relative reflex amplitude as the ratio of the constriction amplitude to the diameter at time 0 multiplied by 100, $\frac{constriction\;amplitude}{diameter\;at\;0\;s}*100$ (Kaltsatou et al., 2011). The duration of constriction is the time interval from the end of latency to the point of maximum constriction (Venkata et al., 2020). On the other hand, the duration of 75% redilation represents the time required for the pupil to recover 75% of the reflex amplitude from its minimum diameter point (Capão Filipe et al., 2003). Redilation amplitude is calculated as the difference between the minimum diameter reached and the diameter measured at the end of the recording (Zhou et al., 2022). This parameter allowed for the calculation of relative total redilation amplitude (redil%) which corresponds to the percentage of recovery-redilation as being the ratio of the redilation amplitude to the constriction amplitude $\frac{diameter\;at\;3.5\;s-minimum\;diameter}{diameter\;at\;0\;s-minimum\;diameter}*100$ (Keivanidou et al., 2010).

#### Kelvin-Voigt model

The Kelvin-Voigt viscoelastic model adapted to photomotor reflex by Yan (Yan et al., 2021) was used to characterize the viscoelastic forces and those related to reflex sympathetic and parasympathetic activities. The balance of forces normalized with respect to the mass of the iris muscles-stroma complex during the photomotor reflex is defined by the Kelvin-Voigt model:

$$\frac{d²r}{dt²}=k_{d2}{\left(l_{0}-r\right)}^{2}+k_{d1}\left(l_{0}-r\right)-D\frac{dr}{dt}-F_{n}\left(t\right)$$ (1)

where $l_{0}$ was defined as the pupil radius equal to the real radius at the start of the signal and r as the pupil radius as a function of time. $k_{d1}$ is a restoring force constant, which is a quantity of force applied over a length in mN/mm and $\left(l_{0}-r\right)$ expresses a length in mm. $k_{d2}$ corresponds to a measure of pressure, a force applied over a surface in mN/mm^2^ and ${\left(l_{0}-r\right)}^{2}$ expresses a surface in mm^2^. The set $k_{d2}{\left(l_{0}-r\right)}^{2}+k_{d1}\left(l_{0}-r\right)$ expresses a restoring force in mN. $D\frac{dr}{dt}$ represents the dynamic viscosity where D is the viscous constant in g/s defined at 4.3 g/s as proposed by Yan (Yan et al., 2021) and $\frac{dr}{dt}$ is a speed in mm/s. This set is expressed in g*mm/s2, which is a viscous force in mN. The force developed by the ANS ( $F_{n}$ ) in mN represents the sum of the forces from the branches of parasympathetic nervous system ( $F_{p}$ ) and sympathetic ( $F_{s}$ ), the force due to the sympathetic system being negative.

$$F_{n}\left(t\right)=F_{p}\left(t\right)-\;F_{s}\left(t\right)$$ (2)

The timing of reflex parasympathetic and sympathetic activations is defined by the following system of equations and allows the identification of the parasympathetic impulse named Impulse 1 ( Imp1 ), the sympathetic impulse named Impulse 3 ( Imp3 ), and the combination of the two named Impulse 2 ( Imp2 ):

$$F_{p}\left(t\right)=f_{p0}*\left[u\left(t-\tau_{p1}\right)-u\left(t-t_{d}-\tau_{p2}\right)\right]$$ (3)

$$F_{s}\left(t\right)=f_{s0}*\left[u\left(t-\tau_{s1}\right)-u\left(t-t_{d}-\tau_{p2}\right)\right]+f_{s1}*\left[u\left(t-t_{d}-\tau_{p2}\right)-u\left(t-t_{d}-\tau_{s2}\right)\right]$$ (4)

$$Imp1=\left(\tau_{s1}-\tau_{p1}\right)*\;f_{p0}$$ (5)

$$Imp2=\left(\tau_{p2}-\tau_{s1}\right)*\left(f_{p0}-f_{s0}\right)$$ (6)

$$Imp3=\left(\tau_{s2}-\tau_{p2}\right)*\;f_{s1}$$ (7)

where $t_{d}$ is the duration of the light stimulus. $f_{p0}$ represents the intensity of the force originating from the parasympathetic nervous system. $\tau_{p1}$ is the delay between the beginning of the light stimulus and the activation of the force $F_{p}$ . $\tau_{p2}$ is the delay between the end of the light stimulus and the end of the force $F_{p}$ . $f_{s0}$ and $f_{s1}$ are used to describe the force $F_{s}$ . The first sympathetic phase ( $f_{s0}$ ) acts in co-activation with the force $f_{p0}$ originating from the parasympathetic nervous system. It is assumed that the first sympathetic phase ( $f_{s0}$ ) always acts with a greater force intensity than the second sympathetic phase ( $f_{s1}$ ). $\tau_{s1}$ is the delay between the beginning of the light stimulus and the activation of the force $F_{s}$ . $\tau_{s2}$ is the delay between the end of the light stimulus and the end of the force $F_{s}$ .

The global Young’s modulus ( $\left.E_{g}\right)$ , (N/m^2^), was calculated from the sum of the restoring forces ( ΣRF ) in N at the moment when the amplitude of the pupil diameter change was maximal, starting from the initial diameter ( ΔR ), in m, the initial length of the material before deformation ( $L_{0}$ ), in m, of the dilator muscle-stroma complex obtained from the edge-to-edge iris diameter minus the average basal pupil diameter of all recordings, and the surface area of the dilator muscle-stroma complex ( A ), in m^2^, averaging 10.5*10^–5^ m^2^ calculated from an iris diameter of 12 mm (Sturm and Larsson, 2009).

$$E_{g}=\frac{\Sigma RF/A}{\Delta R/L_{0}}$$ (8)

The global quality factor of mechanical resonance ( $Q_{g}$ ), unitless, was calculated from F , ΔR , m and D expressed in kilograms per second (kg/s):

$$Q_{g}=\frac{\;\sqrt{\left(\Sigma RF/\Delta R\right)*m}}{D}$$ (9)

Where m expressed in kg, represents the mass of the human wet iris muscles-stroma complex, which may range from 24.4 mg to 26.2 mg depending on its color and shade (Wielgus and Sarna, 2005) averaging 2.53 × 10^−5^ kg.

### Statistical analyses

Evolutionary algorithms, complemented by the classical iterative nonlinear method, were employed to minimize the sum of squares between the Kelvin-Voigt model and the photomotor reflex cycle. This approach was utilized to determine the model parameters that enable a thorough characterization of the reflex parasympathetic and sympathetic activities specific to young elite athletes, taking into consideration the viscoelastic parameters. The coefficient of determination and the mean error were used to characterize the degree of fit of the Kelvin-Voigt model to the photomotor reflex cycle. The normality of the studied variables was tested with a Shapiro-Wilk test. The equality of variances between the studied groups was verified using a Fisher’s test. When the conditions were fulfilled, an independent t-test was used to detect differences in the respective activities of the two branches of the ANS in photomotor reflex between the two groups. A two-dimensional Principal Component Analysis and Pearson’s correlation coefficient were used to highlight linear correlation patterns and to explore the underlying structure of the data by reducing the dimensionality of the variable space studied between the pupillometric parameters and those of HRV. When the conditions for application were not met, a non-parametric Wilcoxon test was performed, and Spearman’s correlation coefficient was used. A threshold of ρ ≤ 0.05 was retained to indicate the significance of the tests. All computations were performed using Rstudio 2022.07.1.

## Results

### Classical description of the photomotor reflex

**FIGURE 2:**
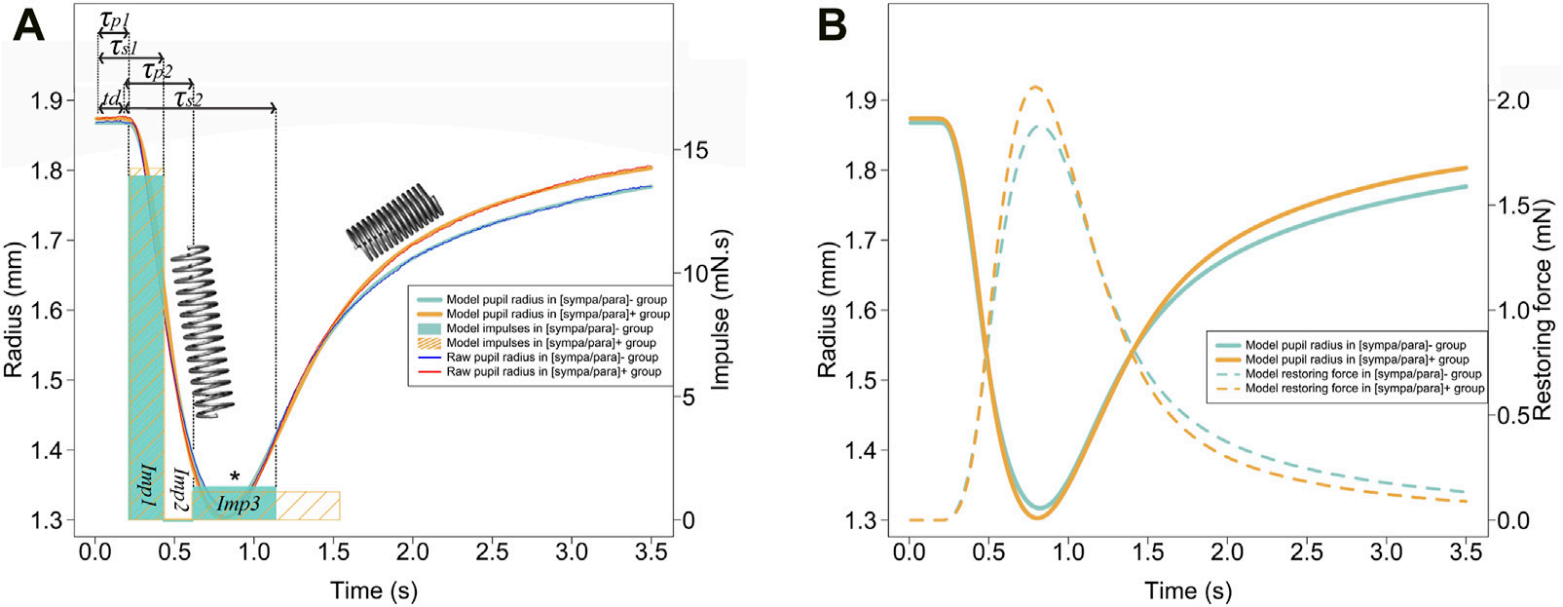
**(A)** The photomotor reflex for each of the two groups is presented with a thin blue line [(sympa/para)- group] and a thin red line (sympa/par + group). The Kelvin-Voigt model for each of the two groups is shown with a thick light blue line [(sympa/para)- group] and a thick orange line [(sympa/para)+ group]. The light blue and orange rectangles represent the impulses of the model for each of the two groups. From left to right, the first rectangles represent impulse 1 (parasympathetic), the second rectangles represent Imp2 (co-activation of both branches of the ANS), and the third rectangles represent Imp3 (sympathetic), which is more significant for the sympa/par + group (*ρ ≤ 0.05). The model parameters were presented only for the [sympa/para]- group to avoid overloading the graph. The spring represents the elastic energy storage-restitution phenomenon of the system, with stretching during the constriction phase and energy restitution during the redilation phase. **(B)** shows the energy storage-restitution through the evolution of the restoring force over time, resulting by the stretching of the dilator muscle-stroma complex induced by the contraction and then relaxation of the constrictor muscle to modify the pupil diameter.

**FIGURE 3:**
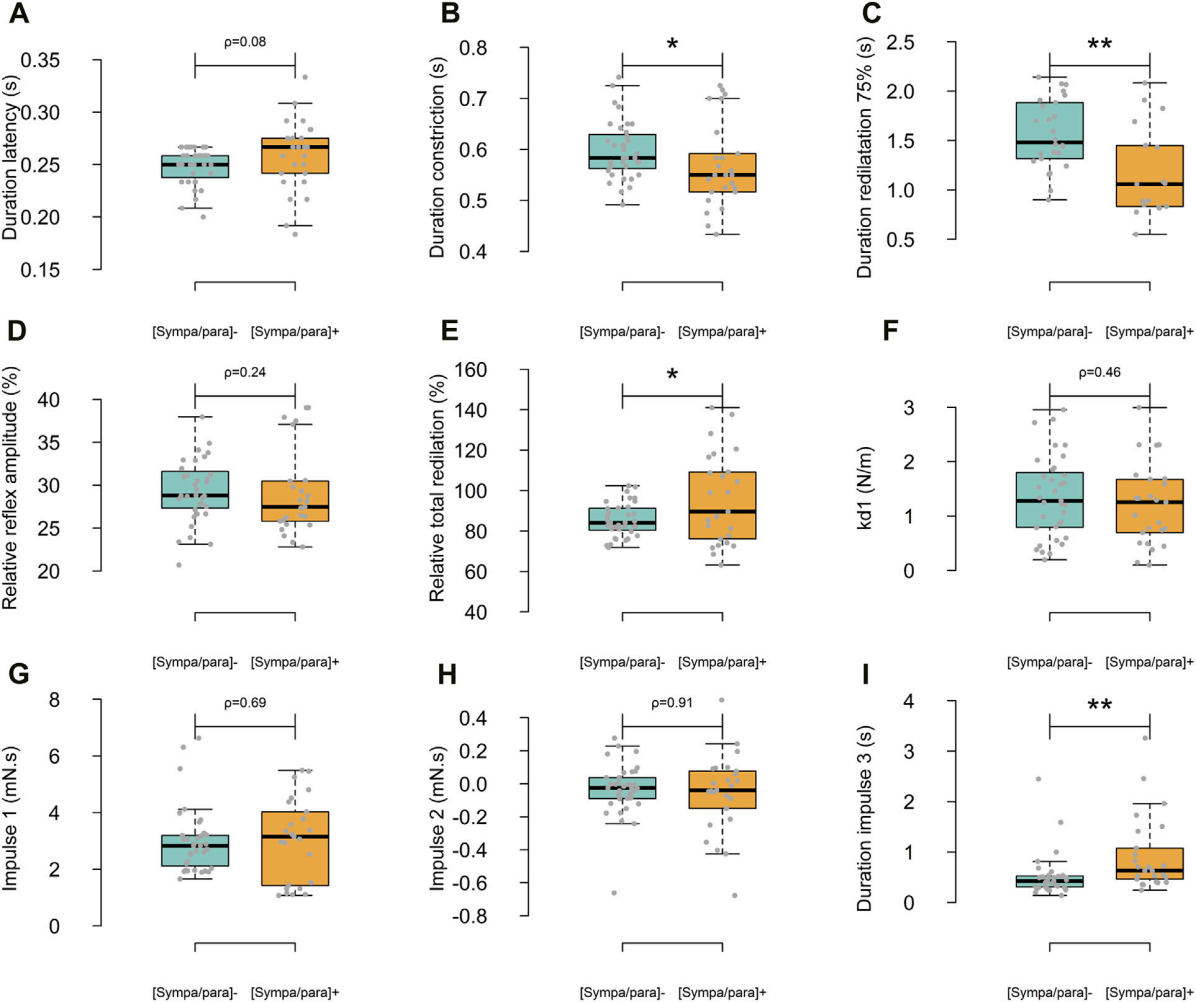
Differences between the two groups regarding the classic descriptive parameters **(A–E)** and those specific to the Kelvin-Voigt model **(F–I)**. In light blue, the [sympa/para]- group (n subjects = 9, n reflexes = 35) and in orange, the [sympa/para]+ group (n subjects = 7, n reflexes = 25). $k_{d1}$ = linear stiffness constant *ρ ≤ 0.05; **ρ ≤ 0.01.

Following the light flash, the duration of latency for all photomotor reflexes averaged 0.253 ± 0.026 s. A constriction phase with a relative amplitude of 29.3% ± 4.3% and a duration of 0.586 ± 0.071 s was observed. The redilation phase, analyzed from the maximum constriction to 3.5 s, lasted 2.570 ± 0.183 s and achieved 89.7% ± 16.5% of the relative total redilation amplitude. The average time to reach maximum constriction speeds across all reflexes was 0.113 ± 0.035 s after the onset of constriction. On the other hand, the average duration from the start of the light flash to reaching 75% of redilation was 2.229 ± 0.463 s. Figure 2A presents the average photomotor reflex of the [sympa/para]^⁻^ and [sympa/para]^+^ groups. The classical descriptive parameters are presented in Figures 3A–E. The duration of constriction and the duration of 75% redilation were significantly longer in the [sympa/para]^⁻^ group compared to the [sympa/para]^+^ group (ρ ≤ 0.05 and ρ ≤ 0.01, respectively). The duration of latency did not show significantly different values between the two groups (ρ = 0.08). The parameter of relative total redilation amplitude was significantly smaller in the [sympa/para]^⁻^ group compared to the [sympa/para]^+^ group (ρ ≤ 0.05). The basal pupil diameter and the parameter of relative reflex amplitude did not vary significantly between the two groups (ρ = 0.58 and ρ = 0.24, respectively). A significant correlation was observed between the duration of constriction and the relative reflex amplitude (r = 0.62, ρ ≤ 0.001).

### Kelvin-Voigt model

The Kelvin-Voigt viscoelastic model perfectly described the 60 photomotor reflexes with a determination coefficient of r^2^ = 0.99 ± 0.01, a sum of squared errors of 5.127 mm^2^, and a mean squared error of 0.085 mm^2^ (Figure 2A). In Figure 2A, the average modeled photomotor reflex of each of the two groups is presented. The model constants are presented in Figures 3F–I. The force parameter related to reflex sympathetic activity, which is the Imp3 (ρ ≤ 0.05), showed significantly different values between the two groups [(sympa/para)^⁻^: 0.55 ± 0.26 mN s, [sympa/para]^+^: 0.76 ± 0.43 mN s] due to a longer duration of Imp3 (D_imp3) (ρ ≤ 0.01) [(sympa/para)^⁻^: 0.521 ± 0.424 s, [sympa/para]^+^: 0.930 ± 0.743 s]. The force of the first sympathetic phase during reflex co-activation ( $f_{s0}$ ) was applied with an intensity of approximately 13.99 ± 4.29 mN in the [sympa/para]^⁻^ group and 14.20 ± 4.83 mN in the [sympa/para]^+^ group. $f_{s1}$ did not show significantly different values between the two groups (ρ = 0.28) [(sympa/para)^⁻^: 1.34 ± 0.78 mN, [sympa/para]^+^: 1.14 ± 0.79 mN]. The force parameters related to reflex parasympathetic activation, which is the Imp1 [(sympa/para)^⁻^: 3.00 ± 1.17 mN, [sympa/para]^+^: 3.04 ± 1.47 mN] and reflex parasympathetic-sympathetic co-activation ( Imp2 ) [(sympa/para)^−^: −0.03 ± 0.16 mN, [sympa/para]^+^: −0.06 ± 0.24 mN] did not show significant differences between the two groups (ρ = 0.69 and ρ = 0.91 respectively). The duration of latency ( $\tau_{p1}$ ) did not show significantly different values between the two groups (ρ = 0.11) [(sympa/para)^−^: 0.211 ± 0.017 s, [sympa/para]^+^: 0.222 ± 0.030 s]. The linear stiffness constant ( $k_{d1}$ ) [(sympa/para)^−^: 1.35 ± 0.75 mN/mm, [sympa/para]^+^: 1.21 ± 0.75 mN/mm] and quadratic stiffness constant ( $k_{d2}$ ) [(sympa/para)^−^: 3.71 ± 2.75 mN/mm^2^, [sympa/para]^+^: 5.85 ± 7.03 mN/mm^2^] did not show significant differences between the two groups (ρ = 0.46 and ρ = 0.95 respectively). The average of ΣRF over all photomotor reflexes was 1.97*10^–3^ ± 1.22*10^–3^ N, ΔR was 0.563*10^–3^ ± 0.258*10^–3^ m. Figure 2B shows that the restoring force reaches its peak at the moment of maximal stretching of the dilator muscle-stroma complex. The initial length ( $L_{0}$ ) of the iris before deformation was 0.324*10^–4^ m.

### Relationship between Kelvin-Voigt model parameters and classical descriptive parameters of the photomotor reflex

**FIGURE 4:**
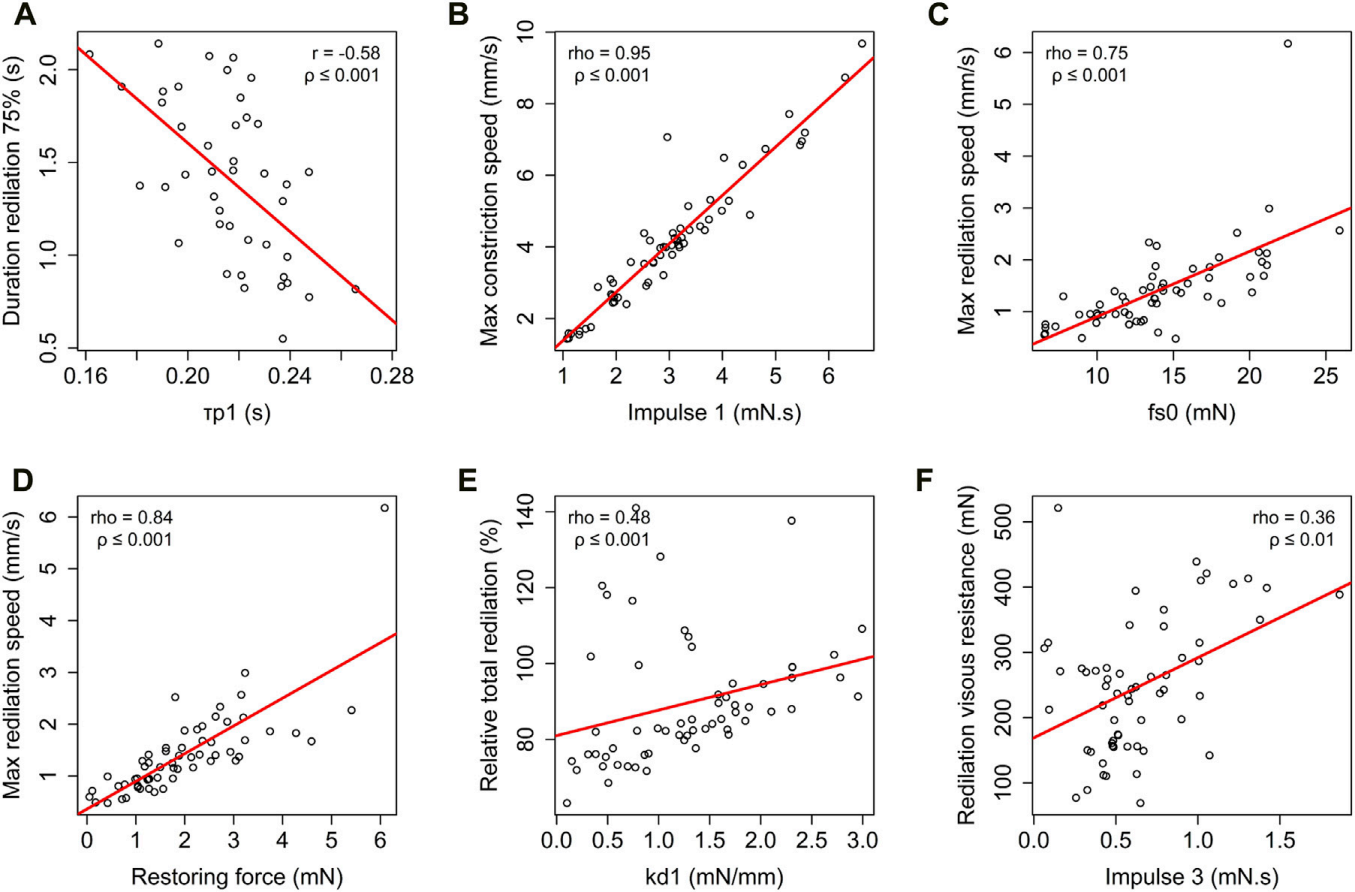
Correlation analysis of descriptive classic photomotor reflex parameters and modelised photomotor reflex parameters: relationship between the duration of 75% redilation and $\tau_{p1}$ **(A)**, between maximum constriction speed and Imp1 **(B)**, between maximum redilation speed and $f_{s0}$ **(C)**, between maximum redilation speed and restoring force **(D)**, between relative total redilation amplitude and linear stiffness constant $k_{d1}$ **(E)** and between redilation viscous resistance and Imp3 **(F)**. The solid line in each panel represents the best-fit line. The correlation coefficient person (r), correlation coefficient spearman (rho), and *p*-value (ρ) are provided to assess the significance of each correlation.

Several significant correlations existed between the model parameters and the classic descriptive parameters of the photomotor reflex (Figure 4). The duration of latency ( $\tau_{p1}$ ) and the duration of 75% redilation were negatively correlated (r = −0.58, ρ ≤ 0.001). The Imp1 was correlated with the maximum constriction speed (rho = 0.95, ρ ≤ 0.001), the Imp2 showed correlations with both the duration of constriction (rho = 0.42, ρ ≤ 0.001) and the relative reflex amplitude (rho = 0.32, ρ ≤ 0.05). The Imp3 exhibited correlations with both the redilation viscous resistance (rho = 0.36, ρ ≤ 0.01) and, negatively, with the duration of latency (rho = −0.31, ρ ≤ 0.05). The duration of Imp3 was negatively correlated with the duration of 75% redilation (rho = −0.48, ρ ≤ 0.001). The maximum redilation speed showed correlations with both the force of the first sympathetic phase during reflex co-activation ( $f_{s0}$ ) (rho = 0.75, ρ ≤ 0.001) and the restoring force (rho = 0.84, ρ ≤ 0.001). The linear stiffness constant ( $k_{d1}$ ) was correlated with the relative total redilation amplitude (rho = 0.48, ρ ≤ 0.05).

### Heart rate variability

**FIGURE 5:**
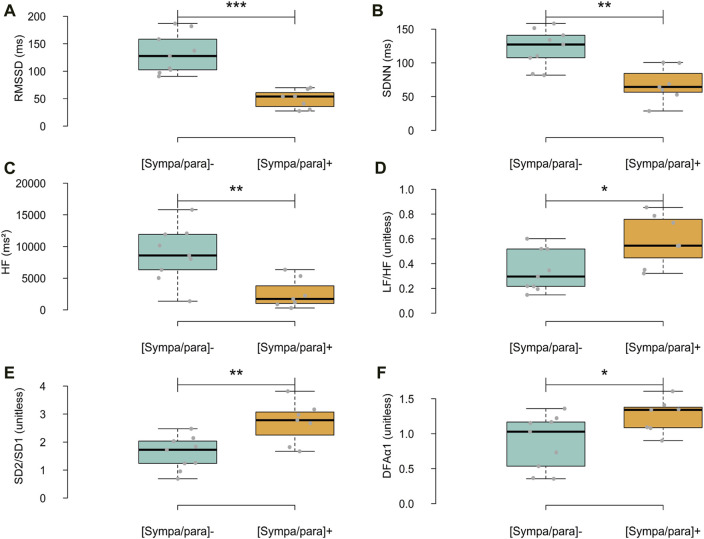
Differences between the two groups regarding HRV parameters in the time domain **(A,B)**, frequency domain **(C,D)**, and non-linear domain **(E,F)**. In light blue, the [sympa/para]- group (n subjects = 9, n analyses = 9) and in orange, the [sympa/para]+ group (n subjects = 7, n analyses = 7). RMSSD = root mean square of successive differences, standard deviation of NN intervals, HF = High frequencies, LF/HF = ratio of low-to high frequency, SD2/SD1 = ratio of standard deviation 2 to standard deviation 1, detrended fluctuation analysis α1 (DFAα1) *ρ ≤ 0.05; **ρ ≤ 0.01; ***ρ ≤ 0.001.

In addition to the HRV parameters used in the dendrogram, Figure 5 presents the differences between the two groups on other major descriptors used in HRV analysis. In the temporal domain, RMSSD and SDNN were significantly higher in the [sympa/para]^⁻^ group than in the [sympa/para]^+^ group (difference of 168.1%, ρ ≤ 0.001 and difference of 79.2%, ρ ≤ 0.01 respectively). In the frequency domain, HF was significantly higher in the [sympa/para]^⁻^ group than in the [sympa/para]^+^ group (difference of 242.6%, ρ ≤ 0.01), and the LF/HF ratio was significantly lower (difference of −42.4% ρ ≤ 0.05). In the non-linear domain, the SD2/SD1 ratio and DFAα1 were significantly lower in the [sympa/para]^-^ group than in the [sympa/para]^+^ group (difference of −40.7% ρ ≤ 0.01 and difference of −29.6% ρ ≤ 0.05 respectively).

### Principal component analysis

The results of the principal component analysis revealed that the first two principal components together explained 60.9% of the total variance. The loadings of the first principal component showed a strong positive association with the variables SD2/SD1: 0.73, Redil%: 0.68, D_imp3: 0.66, and LF/HF: 0.57, and a negative association with RMSSD: −0.84 and HF: −0.67. These results suggest that the first principal component is mainly associated with basal autonomic activity and reflex autonomic activity. The second principal component showed high loadings with Imp1 : −0.82 and $\tau_{p1}$ : 0.64, indicating that it is primarily influenced by reflex parasympathetic activity.

### Correlations between heart rate variability and photomotor reflex

**FIGURE 6:**
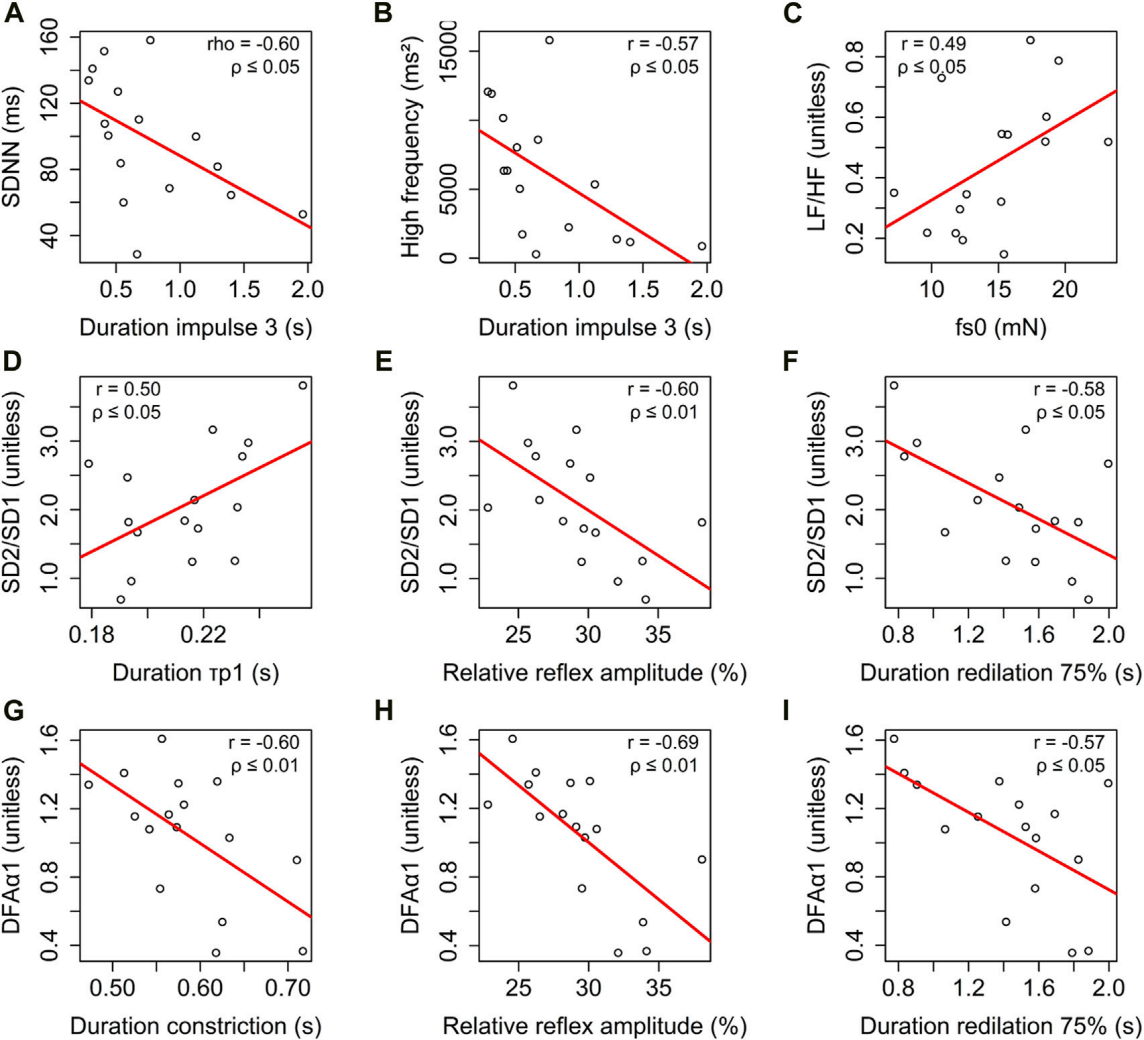
Correlation analysis of heart rate variability parameters across different domains: (i) time domain: relationship between standard deviation of NN intervals (SDNN) and duration of Imp3 **(A)**. (ii) Frequency domain: relationship between high frequency (HF) and duration of Imp3 **(B)**, and between the ratio of low-to high frequencies (LF/HF) and $f_{s0}$ **(C)**. (iii) non-linear domain: relationship between the ratio of SD2 to SD1 and duration of $\tau_{p1}$ **(D)**, relative reflex amplitude **(E)**, and duration of 75% redilation **(F)**. Additionally, the correlation between detrended fluctuation analysis α1 (DFAα1) and duration of constriction **(G)**, relative reflex amplitude **(H)**, and duration of 75% redilation **(I)**. The solid line in each panel represents the best-fit line. The correlation coefficients, Pearson’s (r), Spearman’s (rho), and the *p*-value (ρ) are provided to assess the significance of each correlation.

HRV and the photomotor reflex showed significant correlations (Figure 6). In the temporal domain, SDNN was negatively correlated with the duration of Imp3 (rho = −0.60, ρ ≤ 0.05). In the frequency domain, HF was significantly negatively correlated with the duration of Imp3 (r = −0.57, ρ ≤ 0.05), and the LF/HF ratio was significantly correlated with the force of the first sympathetic phase during reflex co-activation ( $f_{s0}$ ) (r = 0.49, ρ ≤ 0.05). In the non-linear domain, the SD2/SD1 ratio was: positively correlated with $\tau_{p1}$ (r = 0.50, ρ ≤ 0.05), negatively correlated with the relative reflex amplitude (r = −0.60, ρ ≤ 0.05), and negatively correlated with the duration of 75% redilation (r = −0.58, ρ ≤ 0.05). DFAα1 was negatively correlated with: the relative reflex amplitude (r = −0.69, ρ ≤ 0.01), the duration of 75% redilation (r = −0.57, ρ ≤ 0.05), and the duration of constriction (r = −0.60, ρ ≤ 0.05).

### Young’s modulus for the dilator muscle-stroma complex

The Young’s modulus for the dilator muscle-stroma complex, calculated from the restoring forces of all the photomotor reflexes, was 142 N/m^2^.

### Mechanical resonance quality factor

The mechanical resonance quality factor for the restoring force of all the photomotor reflexes was 22.

## Discussion

### Main observations

The Kelvin-Voigt viscoelastic model accurately depicted the constriction-redilation cycle induced by a light signal in a supine position among a population of young elite athletes, as evidenced by the very high determination coefficient (r^2^ = 0.99 ± 0.01) and the very low mean error (MSE = 0.005) obtained (Figure 2A). This perfect description of the photomotor reflex from this model is in line with studies by Yan (Yan et al., 2021) and Fan (Fan and Yao, 2011) conducted on healthy adult participants. The other main observations of our study include (i) characterization of the role of the viscoelastic properties of the iris muscles-stroma complex during the photomotor reflex and the extraction of the precise timing of reflex activity of each of the two branches of the ANS (ii) two groups homogeneous in terms of age, weight, height, and training formed based on three heart rate variability parameters sensitive to the sympathetic/parasympathetic balance also show differences in the parameters extracted from the reflex, suggesting good sensitivity of the latter (iii) the existence of several correlations between parameters characterizing the photomotor reflex and parameters extracted from HRV, both of which inform on the state of the ANS.

### Relationship between photomotor reflex and HRV

**FIGURE 7:**
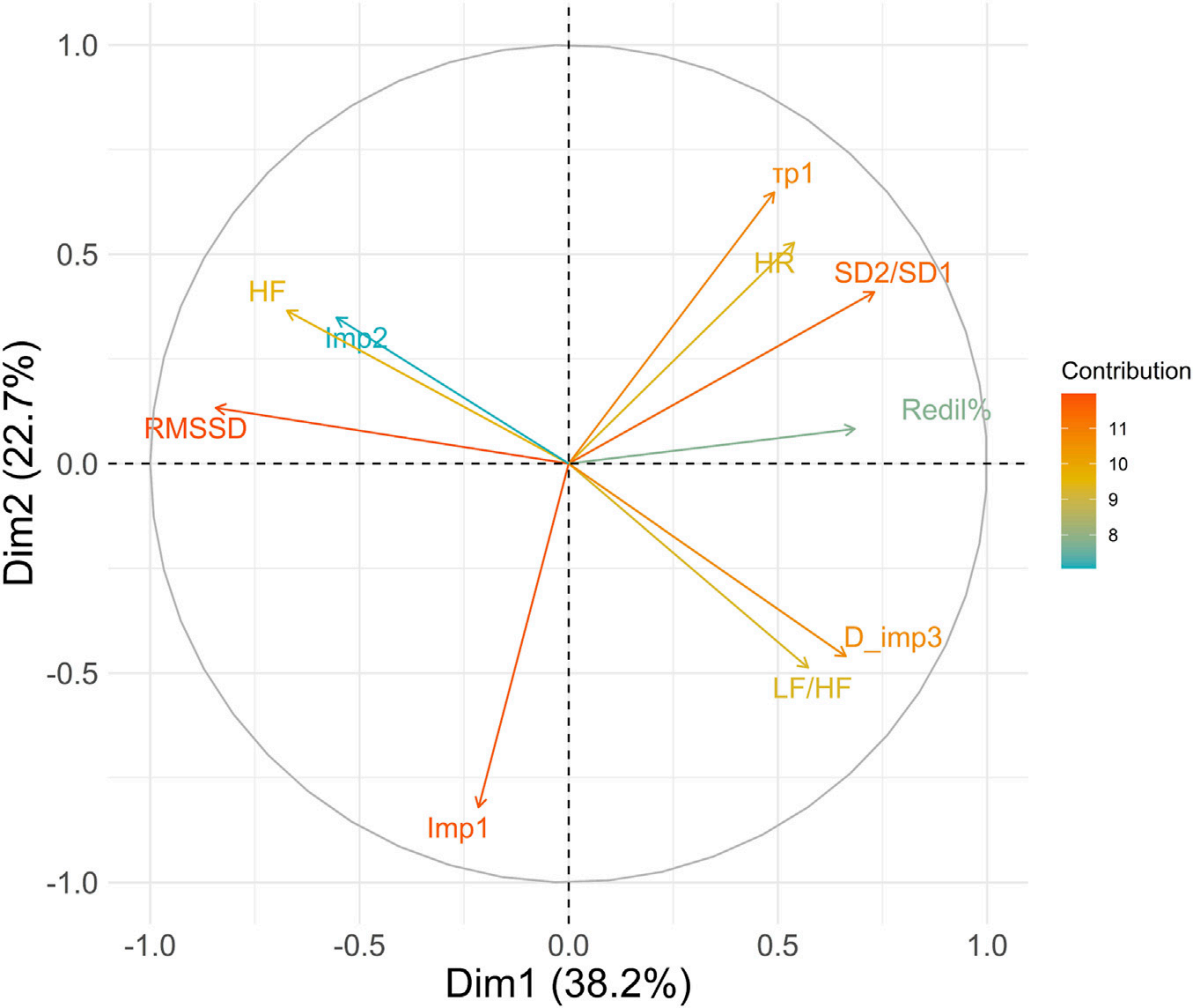
Principal Component Analysis of heart rate variability and photomotor reflex. The color of the vectors applied depends on their contribution to the variance explained by the principal components. Variables with the highest contributions appear in shades closer to red/orange, while those with lower contributions would appear in shades closer to blue. HR, Mean heart rate; RMSSD, root mean square of successive differences; HF, high frequencies; LF/HF, ratio of low-to high frequencies; SD2/SD1, ratio of standard deviation 2 to standard deviation 1; Imp1 , impulse 1; Imp2 , Impulse 2, D_imp3, duration of Imp3 , $\tau_{p1}$ , modelised duration of latency, Redil%, relative total redilation amplitude.

Park (Park et al., 2017) reported very close correlations (r = 0.91 to r = 0.99) between information obtained through spontaneous autonomic activity of the iris muscles and that obtained by HRV, indicating that regardless of the measurement tool applied to different organs, the underlying activity of the ANS is effectively found. The weaker correlations observed between the photomotor reflex and HRV parameters (Figure 6) were anticipated. This outcome aligns with our study’s focus on reflex autonomic activity in response to a stimulus rather than providing direct insights into basal autonomic activity. These findings are consistent with existing literature (Okutucu et al., 2016). In other words, discovering associations and correlations between photomotor reflex activity and basal cardiac activity is expected, given that both are subject to influence from the ANS. However, our results show that the interest of the photomotor reflex probably lies in the complementary information compared to the information that can be obtained through HRV (Figure 5A–F) and spontaneous iris muscles activity. The principal component analysis (Figure 7) reveals the complementarity between basal parasympathetic activity represented by RMSSD and HF and reflex parasympathetic activity represented by the Imp1 (Eqs 3, 5). Imp1 presents an orthogonal orientation with HF and RMSSD, indicating a different meaning between basal parasympathetic activity and reflex parasympathetic activity. This is expected as this parasympathetic reflex impulse ( $\left.Imp1\right)$ constitutes a mechanism for protecting retinal structures and seems to operate independently of basal autonomic activity. Another element of complementarity between basal autonomic activity and reflex autonomic activity is one of the interests of the Kelvin-Voigt model applied to the photomotor reflex is that it allows specifying the nature of the variations between the reflex activity respective of the two branches of the ANS.

### Parasympathetic activity in the photomotor reflex

Regarding reflex parasympathetic activity, Lowenstein and Loewenfeld’s (Lowenstein and Loewenfeld, 1950b) study is a reference for explaining the photomotor reflex. Based on this seminal work, which involved a parasympathectomy by unilateral ciliary ganglion removal in cats, it was demonstrated that the first effective phase of the photomotor reflex, corresponding to constriction, is mainly regulated by the parasympathetic nervous system. Our study is in line with these earlier findings, as we found a correlation between the parasympathetic Imp1 and the maximum constriction speed (Figure 4B). Additionally, a coincidence is noted between reaching maximum speed at 0.113 ± 0.035 s after the start of constriction and the Imp1 (Figure 2A). The Kelvin-Voigt model, adapted by Yan (Yan et al., 2021) (Eq. 1), accurately describes all phases of the photomotor reflex, including the duration of latency, with a reduced average error of 0.036 ± 0.020 s for the parameter $\tau_{p1}$ . Our results are consistent with literature reporting an average duration of latency of 0.250 ± 0.020 s (Bär et al., 2009), and that of our young athletes was almost identical: 0.253 ± 0.026 s. The duration of latency represents the time for transmitting the iris constrictor contraction message through a neural circuit whose main integration levels are the cones and rods providing the afferent message, the pretectal area, the third cranial nerve nuclei, and the ciliary ganglion (Lowenstein and Loewenfeld, 1951; Lowenstein and Loewenfeld, 1959). In addition to purely physical phenomena concerning the length of the neural circuit and the number of connections, it seems that basal autonomic activity can modulate the duration of latency. For instance, the duration of latency decreases with stimulation of basal parasympathetic activity during a 15-min foot immersion in 40°C water (Ishikawa, 2020) or increases with stimulation of basal sympathetic activity in healthy individuals performing a Valsalva maneuver (Gavriysky, 1995). In our study, the negative correlations between the duration of latency and the Imp3 and between the duration of 75% redilation and the duration of Imp3 indicate an influence of sympathetic activity on the duration of latency and the duration of 75% redilation (Figure 4A), in line with the literature (Morley et al., 1991; Gavriysky, 1995). However, our results only show a tendency for difference (*p* = 0.08) between the two groups with distinct basal autonomic activities (Figure 3A). This might be because the aforementioned studies used powerful stimuli from respective branches of the ANS to modulate basal sympathetic activity (Gavriysky, 1995) or basal parasympathetic activity (Ishikawa, 2020) compared to a control situation.

### Parasympathetic and sympathetic co-activation in the photomotor reflex–final constriction phase

Regarding the reflex parasympathetic-sympathetic co-activation (Eq. 2), the constriction phase is attenuated at its end by a reflex sympathetic activation (Capão Filipe et al., 2003) superimposed on the efferent parasympathetic message from the third cranial nerve nucleus (Lowenstein and Loewenfeld, 1950a). The Imp2 of the Kelvin-Voigt model (Eq. 6) characterizes this reflex co-activation of the two ANS branches. In our study, the duration of constriction was significantly shorter in the [sympa/para]^+^ group compared to the [sympa/para]^-^ group (Figure 3B), which aligns with the results of Capão Filipe (Capão Filipe et al., 2003). These authors report a shorter duration of constriction in athletes from strength and explosiveness disciplines compared to endurance runners known for their significant basal parasympathetic activity. The correlation observed in our study between the Imp2 and the duration of constriction is in accordance with the shorter duration of constriction in the [sympa/para]^+^ group compared to the second group. Similarly, the relative reflex amplitude is significantly smaller in subjects such as gymnasts (Capão Filipe et al., 2003), weightlifters (Kaltsatou et al., 2011), or subjects with central serous chorioretinopathy (Zhou et al., 2022), who have less significant basal parasympathetic activity than endurance athletes (Capão Filipe et al., 2003; Kaltsatou et al., 2011) or a healthy control group (Zhou et al., 2022).

### Parasympathetic and sympathetic co-activation in the photomotor reflex–initial redilation phase

Regarding the reflex parasympathetic-sympathetic co-activation (Eq. 2), our results show that the redilation phase in the [sympa/para]^-^ group leads to a longer duration of 75% redilation compared to the [sympa/para]^+^ group (Figure 3C). The Kelvin-Voigt model shows that the duration of 75% redilation is influenced by the reflex co-activation of the parasympathetic and sympathetic nervous systems; the force of the first sympathetic phase during reflex co-activation ( $f_{s0}$ ) being correlated with the maximum redilation speed (Figure 4C) reached during the duration of 75% redilation. This result indicates a significant role of sympathetic reflex activity in the co-activation phase during the first phase of redilation; the force of the first sympathetic phase during co-activation ( $f_{s0}$ ) was 11 times greater than the force of the second sympathetic phase ( $f_{s1}$ ) (Eq. 4) and this was consistent with the study of Yan (Yan et al., 2021). Our results are in line with literature where the ingestion of yohimbine is known to reverse the effect of clonidine and causes a decrease of the duration of 75% redilation (Morley et al., 1991). Yohimbine blocks the action of alpha-2 adrenergic receptors and leads to increased release of norepinephrine and thus to the stimulation of basal sympathetic activity, while clonidine is an activator on alpha-2 adrenergic receptors.

### Sympathetic activity in the photomotor reflex

Regarding reflex sympathetic activity, Lowenstein and Loewenfeld’s studies from 1950 (Lowenstein and Loewenfeld, 1950a; Lowenstein and Loewenfeld, 1950b) on animals show a significant alteration in the dilator muscle’s control of increased pupil diameter in cats that underwent unilateral preganglionic cervical sympathectomy compared to the healthy side. The sympathetic activity is well reflected in our study during the redilation phase, as the Imp3 of the model (Eq. 7), representing reflex sympathetic activity, controls the redilation phase. More specifically, our results show that this reflex sympathetic activation ends in the first third of the redilation phase (Figure 2A), consistent with observations by Yan (Yan et al., 2021). This reflex sympathetic activation, represented by a significantly larger Imp3 (Figure 2A) and larger relative total redilation amplitude in the [sympa/para]^+^ group compared to the [sympa/para]^−^ group (Figure 3E). In the same direction, patients with heart failure (Keivanidou et al., 2010) or central serous chorioretinopathy (Zhou et al., 2022), known for having significant basal sympathetic activity, exhibited a greater relative total redilation amplitude compared to a control group.

### Elastic component and energy restitution in the photomotor reflex

**FIGURE 8:**
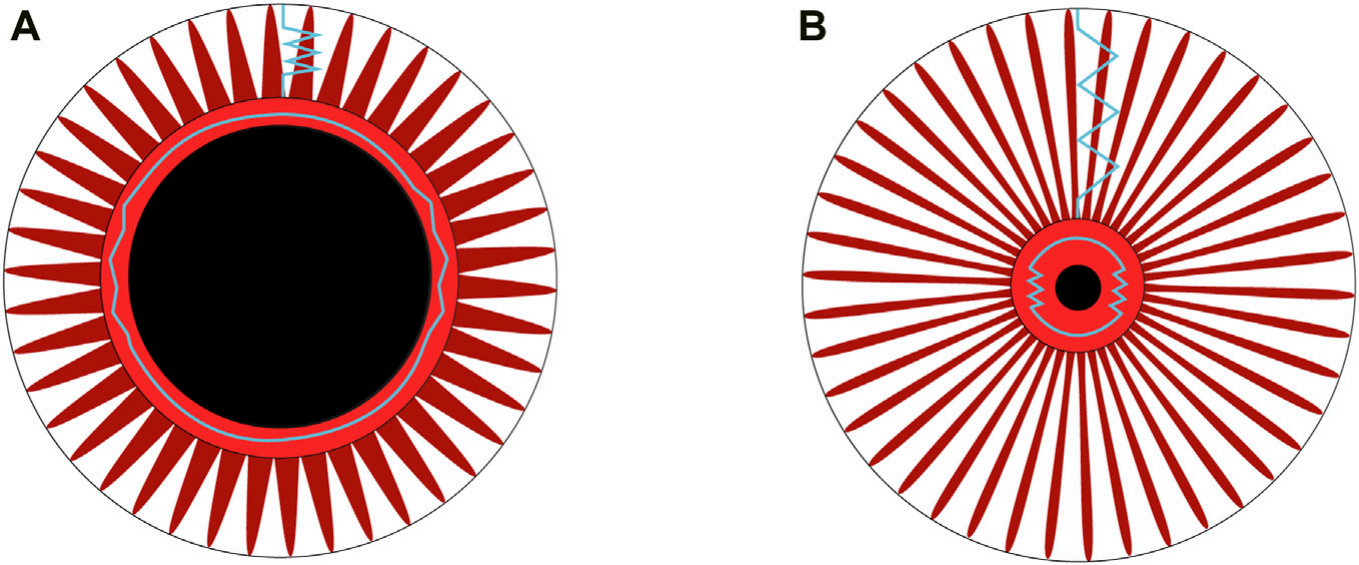
**(A)** The iris muscles-stroma complex before light stimulation. The central black circle represents the dilated pupil, surrounded by the constrictor muscle in a rest condition. Radially arranged around it is the dilator muscle. The blue springs indicate the restoring forces, which are theoretically negligible in a rest condition when the dilator is at its equilibrium length, compared to its tension during the constriction phase of the photomotor reflex cycle. **(B)** After the light flash, the pupil diameter is reduced. The fibers of the dilator muscle, depicted in red and arranged radially, along with the stroma (not shown for simplicity), are stretched, as represented by blue springs. The restoring forces within the dilator muscle-stroma complex reach their maximum when the diameter is at its minimum during the photomotor reflex cycle.

It is interesting to note that the maximum redilation speed was also strongly correlated with the restoring force (Figure 4D) of the model, indicating that redilation is not only under the influence of reflex sympathetic activity but also under the influence of restoring forces. The Imp3 , characterizing reflex sympathetic activation, ends at the final phase of redilation (Figure 2A), and the energy corresponding to this impulse was stored in the elastic components of the dilator muscle-stroma complex and restituted, as evidenced by the correlation between the linear stiffness constant ( $k_{d1}$ ) and the relative total redilation amplitude (Figure 4E). Redilation is therefore influenced by both neural and mechanical parameters. The Kelvin-Voigt model has the merit of revealing the viscoelastic characteristics of the iris muscles-stroma complex (Yan et al., 2021) and highlighting the possibilities of energy storage-restitution mechanisms in the elastic structures of these elements within the constriction-redilation cycle. Based on the Kelvin-Voigt model, we propose that energy storage occurs during the stretching phase of the iris muscles-stroma complex during constriction induced by the light signal (Figure 8). Figure 8 presents a schematic interpretation of the elastic properties described by the Kelvin-Voigt model, applied to the dilator muscle-stroma complex following light stimulation.

Energy restitution occurs during the redilation phase (Figures 2A, B). Narayanaswamy (Narayanaswamy et al., 2019) show that the collagen in the iris muscles-stroma complex, comprising 16% (He et al., 2008), gives it interesting elastic properties according to atomic force microscopy analyses. Considering the constriction followed by redilation in the photomotor reflex, the Kelvin-Voigt model provides a method for measuring the Young’s modulus *in-vivo* of the iris from the sum of restoring forces (Eq. 1). The stiffness obtained through the model (Eq. 8) especially allows for the specification of the dilator muscle-stroma complex, reaching a value of 142 N/m^2^, approaches the lower limit of the range reported in previous studies, which varies from 700 ± 150 N/m^2^ to 38,800 ± 15,800 N/m^2^. Part of this variability can be explained by the different measurement methods used: *ex-vivo* (Narayanaswamy et al., 2019), air-puff optical coherence elastography system (Ye et al., 2021), and optical coherence tomography images after light stimulation of the eye (Pant et al., 2018). The tissues involved in the restitution of stored energy are rich in type I collagen (Silver et al., 2002), are present in the iris (Konstas et al., 1990), have a fibril diameter ranging from 150 to 300 nm (Narayanaswamy et al., 2019), and are 7 times more prevalent than the more compliant type III collagen fibers (He et al., 2008). Collagen, particularly the restoring force plays an important role in a context of natural pupil diameter oscillations with constant iris muscles activity (Bouma and Baghuis, 1971). The restoring force described by the model in our study seems responsible for a large part of the redilation phase, as the reflex sympathetic impulse ( Imp3 ) is relatively brief (0.691 ± 0.608 s) compared to the duration of the redilation phase (2.570 ± 0.183 s) (Figures 2A, B). The observed correlation between the restoring force and the maximum redilation speed (Figure 4D) and between the linear stiffness constant ( $k_{d1}$ ) and the amplitude of the redilation phase (Figure 4E) indeed supports the existence of an elastic energy storage mechanism during the stretching of the dilator muscle-stroma complex during the constriction phase and restitution of this energy in the redilation phase. In our study, the mechanical resonance quality factor of 22 (Eq. 9) suggests the existence of a substantial amount of energy stored and restituted during the constriction-redilation cycle compared to the energy dissipated in the form of damping, a term introduced in the Kelvin-Voigt model in the form of a viscous component (Yan et al., 2021). This viscous component corresponds to a damping function within the constriction-redilation cycle. This damping function is likely related to at least three phenomena: (i) the viscosity of the aqueous humor, which is similar to that of distilled water (Beswick and McCulloch, 1956), surrounding the iris, flowing from the posterior chamber of the eye, and passing through the pupil to enter the anterior chamber (Wang et al., 2016). Specifically, to the intrinsic viscosity of the iris muscles (Fan and Yao, 2011), which is difficult to identify (Sarvazyan et al., 2015), studies often rely on modeled data from the Kelvin-Voigt model. For stable responses of the iris muscles, reported values include 4.30 g/s in healthy subjects (Yan et al., 2021), 3.49 g/s under polychromatic white light of varying color temperatures (from 2,000 K to 10,000 K) and different wavelengths (from 450 nm to 660 nm) (Zandi and Khanh, 2021), and between 3.12 and 3.78 g/s for different lighting conditions (dark or light adaptation) and for varying stimulus intensities (from 10^10.4^ photons/cm^2^/s to 10^12.5^ photons/cm^2^/s) (Fan and Yao, 2011). Hence, it's expected that the Kelvin-Voigt model detects the presence of energy dissipated as heat during muscle contraction (Hill, 1938); (ii) the tissues involved in energy dissipation mechanisms in the form of damping, such as type III collagen (Silver et al., 2002), with fibril diameters ranging from 25 to 100 nm (Narayanaswamy et al., 2019), are present in the iris (Konstas et al., 1990). These energy dissipation mechanisms are necessarily counterbalanced by the reflex sympathetic impulse, which activates the iris dilator muscle to increase the pupil diameter (Figure 4F); (iii) to muscle relaxation mechanisms, which are relatively slow in smooth muscle (Bozler, 1951). Based on work conducted on rodents (Krivoshik and Barr, 2000), it has been shown that the constrictor muscle has a long period of relaxation necessarily associated with a progressively decreasing fraction of myosin heads strongly interacting with actin. This leads to the presence of resistive forces opposing the contraction of the dilator muscle, thus constituting a damping function. This relaxation kinetics, which spans about 10 s (model of isolated constrictor muscle), suggests the existence of a source of energy that can be dissipated when the dilator muscle becomes active against a muscle not yet fully relaxed. In summary, the elastic energy storage-restitution mechanism can be considered a virtuous phenomenon of energy conservation, even if a damping function in the cyclical movement of constriction-redilation is present due to viscosity and slow relaxation kinetics of the constrictor muscle. The main contribution of the Kelvin-Voigt model lies in a better understanding of the viscoelastic properties of the iris muscles that preside in the constriction-redilation cycle. In the same way that the muscle-tendon complexes of the lower limbs are capable of storing energy in the initial phase of foot placement on the ground and restitution in the final phase of thrust during running (Komi, 1984); energy is stored during the constriction phase and partly restituted in the redilation phase, thus allowing very economical cycles of adjustment of the pupil diameter in terms of ATP consumed. The classic descriptive parameters of the photomotor reflex can be directly quantifiable but should be taken with caution as they do not distinguish the forces attributable to reflex sympathetic and parasympathetic activity from those due to viscoelastic forces.

### Limits

One could argue that the LF + HF parameter, which was taken as one of the metrics to distinguish differences in autonomic activity between athletes, is not suitable and that the LF/HF ratio would have been interesting. However, although HF is considered to be purely a reflection of parasympathetic activity, LF does not specifically reflect cardiac sympathetic control in humans (Hopf et al., 1995). Furthermore, the characteristic pattern of the most common state of « fatigue » is a decrease in the total power of HRV (LF + HF) with an increase in heart rate in the supine position compared to a baseline in a non-fatigue condition (Schmitt et al., 2015). These observations are clearly seen in the [sympa/para]+ group compared to the [sympa/para]- group. Otherwise, our experimental setup includes a light stimulation that triggers a retinal protection reflex. In this condition, Impulse one does not represent basal parasympathetic activity but a response to stress. From this perspective, the spontaneous activity formed by hippus could be a solution to capture basal parasympathetic activity. Studies need to be conducted on this subject, even though the signal-to-noise ratio for spontaneous activity is degraded compared to the reflex response.

## Conclusion

In conclusion, the Kelvin-Voigt model applies with great precision to the photomotor reflex of a population of young elite athletes. Beyond its high precision, its main interest lies in its ability to separate information specific to the two branches of the ANS from the viscoelastic properties of the eye, which would otherwise bias the interpretation of classic descriptive parameters. The Kelvin-Voigt model takes into account the physical bases of the constriction-redilation cycle. The high mechanical resonance quality factor observed in our study suggests the existence of an energy recovery mechanism in the constriction-redilation cycle, ensuring significant energy conservation in the continuous cycles of the iris muscles throughout life. The photomotor reflex provides information complementary to HRV analysis, such as the respective characterization of reflex sympathetic and parasympathetic impulses. Within our two groups of athletes, the photomotor reflex allowed us to show that they differ only in their reflex sympathetic impulse. Finally, a practical interest in the daily measurement of the athlete’s ANS activity in response to training loads is highlighted due to the very short duration of photomotor reflex collection of about 3.5 s, which can be compared to the 4 min required for HRV analysis in a supine position.

## Perspectives

In the context of regular measurement of ANS activity along with training loads and performances, the use of the photomotor reflex opens interesting perspectives in monitoring fatigue induced by training load on the autonomic nervous system.The quality of the signal and its reproducibility in athletes allow for the acquisition of three photomotor reflexes of 3.5 s each, spaced 20 s apart (Wang et al., 2018), which permits almost daily measurement to potentially adjust training loads based on their impacts on the ANS. The excellent fit of the Kelvin-Voigt model to the photomotor reflex also provides complementary information to the classical analysis of HRV, as it distinctly appreciates the respective activities of the two branches of the ANS, distinguishing them from the part of the reflex attributed to mechanical aspects. The Kelvin-Voigt model is capable of detecting subtle differences in sympathetic nervous system activity between the two groups of athletes formed based on the balance between the two branches of the autonomic nervous system initially evaluated with heart rate variability. Among the practical applications currently being developed for use by a wide range of athletes, a version of the device is being adapted for smartphones and will need to undergo a validation process against a reference system.

## Data Availability

The raw data supporting the conclusions of this article will be made available by the authors, without undue reservation.

## References

[B1] Abokyi S. Owusu-Mensah J. Osei K. A. (2017). Caffeine intake is associated with pupil dilation and enhanced accommodation. Eye (Basingstoke) 31, 615–619. 10.1038/eye.2016.288 PMC5396006 27983733

[B2] Bär K. J. J. Schulz S. Koschke M. Harzendorf C. Gayde S. Berg W. (2009). Correlations between the autonomic modulation of heart rate, blood pressure and the pupillary light reflex in healthy subjects. J. Neurol. Sci. 279, 9–13. 10.1016/j.jns.2009.01.010 19195664

[B3] Beckers F. Verheyden B. Aubert A. E. (2006). Aging and nonlinear heart rate control in a healthy population. Am. J. Physiol. Heart Circ. Physiol. 290, H2560–H2570. 10.1152/ajpheart.00903.2005 16373585

[B4] Beswick J. A. McCulloch C. (1956). Effect of hyaluronidase on the viscosity of the aqueous humour. Br. J. Ophthalmol. 40, 545–548. 10.1136/bjo.40.9.545 13364181 PMC1324678

[B5] Bhowmik S. Arjunan S. P. Sarossy M. Radcliffe P. J. Kumar D. K. (2021). Pupillometric recordings to detect glaucoma. Physiol. Meas. 42 (4), 045003. 10.1088/1361-6579/abf05c 33740779

[B6] Bouma H. Baghuis L. C. J. (1971). Hippus of the pupil: periods of slow oscillations of unknown origin. Vis. Res. 11, 1345–1351. 10.1016/0042-6989(71)90016-2 5148578

[B7] Bourdillon N. Schmitt L. Yazdani S. Vesin J. M. Millet G. P. (2017). Minimal window duration for accurate HRV recording in athletes. Front. Neurosci. 11, 456. 10.3389/fnins.2017.00456 28848382 PMC5554345

[B8] Bourdillon N. Yazdani S. Vesin J. M. Schmitt L. Millet G. P. (2022). RMSSD is more sensitive to artifacts than frequency-domain parameters: implication in athletes’ monitoring. J. Sports Sci. Med. 21, 260–266. 10.52082/jssm.2022.260 35719238 PMC9157524

[B9] Bozler E. (1951). Mechanism of relaxation in extracted muscle fibers. Am. J. Physiol. 167, 276–283. 10.1152/ajplegacy.1951.167.1.276 14885497

[B10] Capão Filipe J. A. Falcão-Reis F. Castro-Correia J. Barros H. (2003). Assessment of autonomic function in high level athletes by pupillometry. Auton. Neurosci. 104, 66–72. 10.1016/s1566-0702(02)00268-0 12559205

[B11] Cuve H. C. Stojanov J. Roberts-Gaal X. Catmur C. Bird G. (2022). Validation of Gazepoint low-cost eye-tracking and psychophysiology bundle. Behav. Res. Methods 54, 1027–1049. 10.3758/s13428-021-01654-x 34405387 PMC9046335

[B12] Ehinger B. V. Groß K. Ibs I. König P. (2019). A new comprehensive eye-tracking test battery concurrently evaluating the Pupil Labs glasses and the EyeLink 1000. PeerJ 7, e7086. 10.7717/peerj.7086 31328028 PMC6625505

[B13] Fan X. Yao G. (2011). Modeling transient pupillary light reflex induced by a short light flash. IEEE Trans. Biomed. Eng. 58, 36–42. 10.1109/TBME.2010.2080678 20876003 PMC4318650

[B14] Gavriysky V. S. (1995). Human pupillary light reflex during and after two-fold Valsalva maneuver. J. Auton. Nerv. Syst. 54, 247–252. 10.1016/0165-1838(95)00017-r 7490426

[B15] Guzik P. Piskorski J. Krauze T. Schneider R. Wesseling K. H. Wykretowicz A. (2007). Correlations between the Poincaré plot and conventional heart rate variability parameters assessed during paced breathing. J. Physiol. Sci. 57, 63–71. 10.2170/physiolsci.RP005506 17266795

[B16] He M. Lu Y. Liu X. Ye T. Foster P. J. (2008). Histologic changes of the iris in the development of angle closure in Chinese eyes. J. Glaucoma 17, 386–392. 10.1097/IJG.0b013e31815c5f69 18703949

[B17] Heart rate Variability (1996). Standards of measurement, physiological interpretation, and clinical use. *Task Force of the European Society of Cardiology and the North American Society of Pacing and Electrophysiology* . Circulation 93, 1043–1065. 10.1161/01.cir.93.5.1043 8598068

[B18] Heys J. Barocas V. H. (1999). Mechanical characterization of the bovine iris. J. Biomech. 32, 999–1003. 10.1016/s0021-9290(99)00075-5 10460139

[B19] Hill A. V. (1938). The heat of shortening and the dynamic constants of muscle. Proc. Roy. Soc. Lond B 126, 136–195. 10.1098/rspb.1938.0050

[B20] Hopf H. B. Skyschally A. Heusch G. Peters J. (1995). Low-frequency spectral power of heart rate variability is not a specific marker of cardiac sympathetic modulation. Anesthesiology 82, 609–619. 10.1097/00000542-199503000-00002 7879929

[B21] Ishikawa M. (2020). Effective combination of pupil light reflex and heart rate variability to assess foot bath effects on autonomic function in healthy adults. Biomed. Phys. Eng. 6 (1), 015034. 10.1088/2057-1976/ab6e1c 33438622

[B22] Kaltsatou A. Kouidi E. Fotiou D. Deligiannis P. (2011). The use of pupillometry in the assessment of cardiac autonomic function in elite different type trained athletes. Eur. J. Appl. Physiol. 111, 2079–2087. 10.1007/s00421-011-1836-0 21259023

[B23] Karlsen R. L. Søli N. (1979). Changes in pupillary dynamics in young men during prolonged severe exercise. Acta Ophthalmol. 57, 41–47. 10.1111/j.1755-3768.1979.tb06657.x 419975

[B24] Keivanidou A. Fotiou D. Arnaoutoglou C. Arnaoutoglou M. Fotiou F. Karlovasitou A. (2010). Evaluation of autonomic imbalance in patients with heart failure: a preliminary study of pupillomotor function. Cardiol. J. 17, 65–72. 20104459

[B25] Komi P. V. (1984). Physiological and biomechanical correlates of muscle function. Exerc Sport Sci. Rev. 12, 81–122. 10.1249/00003677-198401000-00006 6376140

[B26] Konstas A. G. Marshall G. E. Lee W. R. (1990). Immunocytochemical localisation of collagens (I–V) in the human iris. Graefes Arch. Clin. Exp. Ophthalmol. 228, 180–186. 10.1007/BF00935730 2186974

[B27] Krivoshik A. P. Barr L. (2000). Force relaxes before the fall of cytosolic calcium in the photomechanical response of rat sphincter pupillae. Am. J. Physiol. Cell. Physiol. 279, C274–C280. 10.1152/ajpcell.2000.279.1.C274 10898739

[B28] Longtin A. Milton J. G. (1989). Modelling autonomous oscillations in the human pupil light reflex using non-linear delay-differential equations. Bull. Math. Biol. 51, 605–624. 10.1007/BF02459969 2804468

[B29] Lowenstein O. Loewenfeld I. E. (1950a). Mutual role of sympathetic and parasympathetic in shaping of the pupillary reflex to light: pupillographic studies. Arch. Neurol. Psychiatry 64, 341–377. 10.1001/archneurpsyc.1950.02310270030002 15433652

[B30] Lowenstein O. Loewenfeld I. E. (1950b). Role of sympathetic and parasympathetic systems in reflex dilatation of the pupil: pupillographic Studies. Arch. Neurol. Psychiatry 64, 313–340. 10.1001/archneurpsyc.1950.02310270002001 15433651

[B31] Lowenstein O. Loewenfeld I. E. (1951). Types of central autonomic innervation and fatigue: pupillographic studies. Arch. Neurol. Psychiatry 66, 580–599. 10.1001/archneurpsyc.1951.02320110045004 14868027

[B32] Lowenstein O. Loewenfeld I. E. (1959). Scotopic and photopic thresholds of the pupillary light reflex in normal man. Am. J. Ophthalmol. 48, 87–98. 10.1016/0002-9394(59)90245-4 13670267

[B33] Morley M. Bradshaw C. Szabadi E. (1991). Effects of clonidine and yohimbine on the pupillary light reflex and carbachol‐evoked sweating in healthy volunteers. Br. J. Clin. Pharmacol. 31, 99–101. 10.1111/j.1365-2125.1991.tb03864.x 2015177 PMC1368419

[B34] Narayanaswamy A. Nai M. H. Nongpiur M. E. Htoon H. M. Thomas A. Sangtam T. (2019). Young’s modulus determination of normal and glaucomatous human Iris. Investig. Ophthalmol. Vis. Sci. 60, 2690–2695. 10.1167/iovs.18-26455 31242291

[B35] Okutucu S. Civelekler M. Aparci M. Sabanoglu C. Dikmetas O. Aksoy H. (2016). Computerized dynamic pupillometry indices mirrors the heart rate variability parameters. Eur. Rev. Med. Pharmacol. Sci. 20, 2099–2105. 27249610

[B36] Pant A. D. Gogte P. Pathak-Ray V. Dorairaj S. K. Amini R. (2018). Increased iris stiffness in patients with a history of angle-closure glaucoma: an image-based inverse modeling analysis. Investig. Ophthalmol. Vis. Sci. 59, 4134–4142. 10.1167/iovs.18-24327 30105368

[B37] Park S. Won M. J. Lee D. W. Whang M. (2017). Non-contact measurement of heart response reflected in human eye. Int. J. Psychophysiol. 123, 179–198. 10.1016/j.ijpsycho.2017.07.014 28757234

[B38] Perrotta A. S. Jeklin A. T. Hives B. A. Meanwell L. E. Warburton D. E. R. (2017). Validity of the elite hrv smartphone application for examining heart rate variability in a field-based setting. J. Strength Cond. Res. 31, 2296–2302. 10.1519/JSC.0000000000001841 28195974

[B39] Pichot V. Roche F. Gaspoz J. M. Enjolras F. Antoniadis A. Minini P. (2000). Relation between heart rate variability and training load in middle-distance runners. Med. Sci. Sports Exerc. 32, 1729–1736. 10.1097/00005768-200010000-00011 11039645

[B40] Saboul D. Pialoux V. Hautier C. (2012). The breathing effect of the LF/HF ratio in the heart rate variability measurements of athletes. Eur. J. Sport Sci. 14 (Suppl. 1), S282–S288. 10.1080/17461391.2012.691116 24444219

[B41] Sarvazyan A. Tsyuryupa S. Rudenko O. (2015). Ability of skeletal muscle to protect bones and joints from external impacts: acoustical assessment. J. Acoust. Soc. Am. 137, 2425. 10.1121/1.4920847

[B42] Schmitt L. Bouthiaux S. Millet G. P. (2021). Eleven years’ monitoring of the world’s most successful male biathlete of the last decade. Int. J. Sports Physiol. Perform. 16, 900–905. 10.1123/ijspp.2020-0148 32887848

[B43] Schmitt L. Regnard J. Parmentier A. L. Mauny F. Mourot L. Coulmy N. (2015). Typology of fatigue by heart rate variability analysis in elite nordic-skiers. Int. J. Sports Med. 36, 999–1007. 10.1055/s-0035-1548885 26252552

[B44] Silver F. H. Horvath I. Foran D. J. (2002). Mechanical implications of the domain structure of fiber-forming collagens: comparison of the molecular and fibrillar flexibilities of the alpha1-chains found in types I-III collagen. J. Theor. Biol. 216, 243–254. 10.1006/jtbi.2002.2542 12079374

[B45] Stark L. Sherman P. M. (1957). A servoanalytic study of consensual pupil reflex to light. J. Neurophysiol. 20, 17–26. 10.1152/jn.1957.20.1.17 13398849

[B46] Sturm R. A. Larsson M. (2009). Genetics of human iris colour and patterns. Pigment. Cell. Melanoma Res. 22, 544–562. 10.1111/j.1755-148X.2009.00606.x 19619260

[B47] Usui S. Hirata Y. (1995). Estimation of autonomic nervous activity using the inverse dynamic model of the pupil muscle plant. Ann. Biomed. Eng. 23, 375–387. 10.1007/BF02584438 7486345

[B48] Venkata S. A. Kalburgi-Narayana M. Kuppusamy M. Ramaswamy P. Bachali S. (2020). Computerized dynamic pupillometry as a screening tool for evaluation of autonomic activity. Neurophysiol. Clin. 50, 321–329. 10.1016/j.neucli.2020.09.004 33051091

[B49] Wang W. Qian X. Song H. Zhang M. Liu Z. (2016). Fluid and structure coupling analysis of the interaction between aqueous humor and iris. Biomed. Eng. Online 15 (Suppl. 2), 133. 10.1186/s12938-016-0261-3 28155692 PMC5260046

[B50] Wang Y. Zekveld A. A. Wendt D. Lunner T. Naylor G. Kramer S. E. (2018). Pupil light reflex evoked by light-emitting diode and computer screen: Methodology and association with need for recovery in daily life. PLoS ONE 13 (6), e0197739. 10.1371/journal.pone.0197739 29897946 PMC5999086

[B51] Wielgus A. R. Sarna T. (2005). Melanin in human irides of different color and age of donors. Pigment. Cell. Res. 18, 454–464. 10.1111/j.1600-0749.2005.00268.x 16280011

[B52] Wilson M. H. Edsell M. Imray C. Wright A. Birmingham Medical Research Expeditionary Society (2008). Changes in pupil dynamics at high altitude - an observational study using a handheld pupillometer. High. Alt. Med. Biol. 9, 319–325. 10.1089/ham.2008.1026 19115917

[B53] Yamaji K. Yoshitomi T. Usui S. Ohnishi Y. (2003). Mechanical properties of the rabbit iris smooth muscles. Vis. Res. 43, 479–487. 10.1016/s0042-6989(02)00574-6 12536003

[B54] Yan Y. J. Chen C. N. Ou-Yang M. (2021). Using system identification to construct an inherent model of pupillary light reflex to explore diabetic neuropathy. Brain Sci. 11, 852. 10.3390/brainsci11070852 34202410 PMC8301861

[B55] Ye S. Zhou Y. Bao C. Chen Y. Lu F. Zhu D. (2021). *In vivo* non-contact measurement of human iris elasticity by optical coherence elastography. J. Biophot. 14 (9), e202100116. 10.1002/jbio.202100116 34051066

[B56] Zandi B. Khanh T. Q. (2021). Deep learning-based pupil model predicts time and spectral dependent light responses. Sci. Rep. 11 (1), 841. 10.1038/s41598-020-79908-5 33436693 PMC7803766

[B57] Zhou X. Fukuyama H. Okita Y. Kanda H. Yamamoto Y. Araki T. (2022). Pupillary responses reveal autonomic regulation impairments in patients with central serous chorioretinopathy. Investig. Ophthalmol. Vis. Sci. 63 (10), 2. 10.1167/iovs.63.10.2 PMC9463716 36066317

